# nbCNV: a multi-constrained optimization model for discovering copy number variants in single-cell sequencing data

**DOI:** 10.1186/s12859-016-1239-7

**Published:** 2016-09-17

**Authors:** Changsheng Zhang, Hongmin Cai, Jingying Huang, Yan Song

**Affiliations:** School of Computer Science & Engineering, South China University of Technology, Guangzhou, 510006 China

**Keywords:** Copy number variants, Read depth, Negative binomial distribution, Sparsity, Smoothness, ADMM

## Abstract

**Background:**

Variations in DNA copy number have an important contribution to the development of several diseases, including autism, schizophrenia and cancer. Single-cell sequencing technology allows the dissection of genomic heterogeneity at the single-cell level, thereby providing important evolutionary information about cancer cells. In contrast to traditional bulk sequencing, single-cell sequencing requires the amplification of the whole genome of a single cell to accumulate enough samples for sequencing. However, the amplification process inevitably introduces amplification bias, resulting in an over-dispersing portion of the sequencing data. Recent study has manifested that the over-dispersed portion of the single-cell sequencing data could be well modelled by negative binomial distributions.

**Results:**

We developed a read-depth based method, *nbCNV* to detect the copy number variants (CNVs). The nbCNV method uses two constraints-sparsity and smoothness to fit the CNV patterns under the assumption that the read signals are negatively binomially distributed. The problem of CNV detection was formulated as a quadratic optimization problem, and was solved by an efficient numerical solution based on the classical alternating direction minimization method.

**Conclusions:**

Extensive experiments to compare nbCNV with existing benchmark models were conducted on both simulated data and empirical single-cell sequencing data. The results of those experiments demonstrate that nbCNV achieves superior performance and high robustness for the detection of CNVs in single-cell sequencing data.

**Electronic supplementary material:**

The online version of this article (doi:10.1186/s12859-016-1239-7) contains supplementary material, which is available to authorized users.

## Background

Copy number variants (CNVs), which constitute a major form of DNA structural variation, have been shown to be closely related to several diseases, including autism [11], schizophrenia [29] and cancer [5, 8, 15, 21]. Comparative genomic hybridization and fluorescence in situ hybridization have been used to detect CNVs of particular genes or fragments [26] but are limited in terms of resolution. To profile genome-wide copy number (CN) landscapes, these techniques have consequently been replaced by next-generation sequencing (NGS) technologies [9]. Because it uses bulk DNA from tissue samples, however, traditional sequencing provides an average signal from millions of cells and is thus of limited utility for the characterization of tumor heterogeneity at the single-cell level.

An innovative technique, single-cell sequencing (SCS), was developed to address key issues in cancer studies, including measurement of mutation rates, tracing of cell lineages, resolution of intra-tumor heterogeneity and elucidation of tumor evolution [21, 22]. SCS combines flow sorting of single cells, whole-genome amplification (WGA) and NGS to characterize the genome-wide CN of single cells. Existing WGA techniques, such as degenerate oligonucleotide primed-polymerase chain reaction [30], multiple displacement amplification [17] and multiple annealing looping-based amplification cycling [36], inevitably introduce amplification bias to varying degrees when the whole genome of a single cell is amplified to microgram levels for NGS [13, 28]. Such technical noise due to amplification bias is over-dispersed and is different from that of bulk sequencing, which does not involve amplification. There are two main strategies that use NGS data to detect CNVs: read depth (RD)-based and read pair (RP)-based methods [20]. To the best of our knowledge, RD-based methods are arguably popular for CNV detection. Furthermore, CNV detection using SCS data requires only sparse sequence coverage to economically accommodate numerous single cells [4].

The analysis pipeline of RD-based methods consists of data preparation (optional), data normalization (optional), CNV region identification (core) and CN profile estimation (optional) [19]. Briefly speaking, the reference genome is divided into equally or variably sized, non-overlapping bins for computing read counts (RCs) in each bin along the whole genome. The RD in each bin is generated by the corresponding RC divided by the average RC for the whole genome. The RD signal is then normalized using strategies such as lowness smoothing based on guanine-cytosine (GC) content. Different segmentation algorithms are used to detect the CNV regions. After their detection, the CNV regions can be translated into a CN profile using available ploidy information or by other methods [3, 19]. Several existing RD-based benchmark CNV detection methods, which we later apply for comparison, are described as follows.

DNAcopy [27] implements a classical circular binary segmentation (CBS) [25] algorithm to segment RD data and identifies abnormal genomic regions. The basic idea of CBS is to translate a noisy-intensity RD signal into regions of equal CNs followed by binary segmentation. Copynumber [24] is a highly efficient algorithm that offers a unified framework to segment RD data from single or multiple samples. This approach combines least squares principles with a suitable penalization scheme for a given number of breakpoints to detect CN profiles. The above two methods do not require data preparation and normalization. Control-free copy number and allelic content caller (Control-FREEC) [6] is a systematic CNV detection package consisting of data preparation, normalization, CNV region identification, and profile estimation. Control-FREEC segments the whole reference genome into equally sized, non-overlapping bins. It then computes the RD of the tested sample in each bin. If a control sample is not supplied, Control-FREEC uses the GC content in each bin to achieve data normalization. For CNV detection, Control-FREEC uses least absolute shrinkage and selection operator (LASSO) regression. CNVnator [1] also divides the whole reference genome (hg18 or hg19) into continuous, non-overlapping equal-sized bins. Normalization is achieved by averaging the RD signal over each bin with respect to GC content, and CNV region identification is based on mean-shift tracking.

**Fig. 1:**
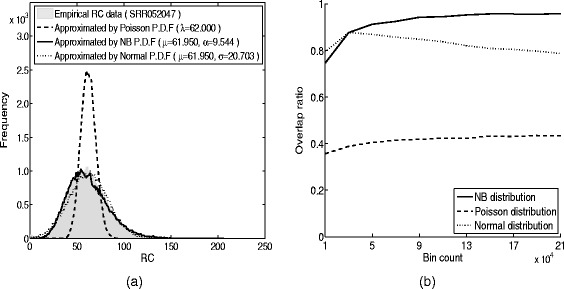
Modeling of single-cell sequencing RC data and selection of a suitable bin count for experiments. **a** Frequency histogram of RC data (accession number SRR052047), shown in light green. For comparison, the frequencies of simulated RCs generated by Poisson, Gaussian and negative binomial distributions are indicated by brown, yellow or dark green lines, respectively. The RCs of the SCS data can be accurately characterized by a negative binomial distribution. The estimated parameters by MLE for each P.D.F are shown in parenthesis. **b** Relationship between bin count (horizontal axis) and overlap ratio (vertical axis). The empirical sequencing data could be better approximated by a negative binomial distribution. Such nice approximation was insensitive to the bin count. Therefore, the proposed nbCNV is hoping to be applied in various bin count setting

Most of the earlier analysis in sequencing analysis assumed that the RDs are following Gaussian distribution [2, 24, 31] or Poisson distribution [1, 10, 14]. However, experimental analysis in the absolute numbers of mRNA molecules by single-cell sequencing manifested [12] that the counts could be accurately characterized by negative binomial distribution. To this aim, we firstly generated an illustrative example to characterize the statistical distribution of real RDs by single-cell sequencing technology. Real sequencing data from a normal cell (accession number SRR052047) [21] were preprocessed to obtain RC data with 50000 variable bins. The frequency histogram of the RC data is shown in Fig. 1(a). The distribution was approximated by the Poisson, Gaussian and negative binomial probability density functions through maximum likelihood estimation (MLE). The estimated mean value of the Poisson distribution was *λ*=62. For the negative binomial distribution, the estimated mean value and dispersion coefficient are *μ*=61.94 and *α*=9.544, respectively. For Gaussian distribution, the estimated mean value was *μ*=61.950 and the estimated standard deviation *σ*=20.703. This figure clearly demonstrated that the frequency histogram of the real RC data could be nicely characterized by a negative binomial distribution. For further comparison, we also measured the overlap ratio between the real RC and its approximations with different bin counts, ranging from 10000 to 210000. The overlap ratio was calculated as,

1 $$ f(x) = \frac{A(x)\bigcap B(x)}{A(x)\bigcup B(x)}  $$

where *A* denotes the frequency of empirical RC *x* and *B* is the approximated probability density function. The result was shown in Fig. 1(b). The ratio between the empirical RC and the one approximated by NB distribution was dramatically higher than by the Poisson distribution. When the bin count was larger than 50000, the ratio is more than 0.9 by the NB distribution. In comparison, the ratio was low to 0.4 by Poisson distribution. For Gaussian distribution, the ratio was rising when the bin count ranged from 10000 to 30000 and the ratio under 30000 was as higher as the one by NB distribution. However, it began dropping continuously with the bin count rising continuously. Therefore, the RC distribution could be accurately characterized by the negative binomial distribution.

To this end, a novel model called *nbCNV* was proposed in this paper to detect CNVs using SCS data. The nbCNV model uses negative binomial distributions to approximate loci along the whole genome. We incorporate two constraints of sparsity and smoothness to fit the CNV patterns. The CNV detection problem is then formulated by a quadratic optimization model. The proposed nbCNV uses an efficient numerical scheme based on the classical alternating direction minimization method (ADMM) to achieve efficiency. Since SCS data analysis requires carefully data preprocessing, a recently published SCS protocol [3] was modified to fit with the proposed nbCNV detection method. We have built a systematical pipeline for single-cell sequencing analysis. Considering the inherent contradiction between the quality [7] and resolution of CNVs detected with RD-based methods, nbCNV can adaptively select the most suitable total number of bins according to user preference. Once ploidy information is provided, the CNV regions detected by nbCNV can be translated into a CN profile.

The rest of this article is organized as follows. The underling mathematical models of nbCNV and its numerical solution are described in section “Methods”. We then evaluate and demonstrate the efficiency of nbCNV compared with several benchmark methods using both simulated and real SCS datasets in section “Results and discussion”. Finally, we conclude the paper in section “Conclusions”.

## Methods

### Data preprocessing

To achieve data preparation and normalization, we modified a previously reported protocol for genome-wide CN analysis of single cells [3]. The steps are briefly summarized here. We first downloaded a sequencing file from the National Center for Biotechnology Information (NCBI) short read archive (SRA) and used the bowtie2 alignment tool [16] to map the millions of short reads to the GRCh37 human reference genome. Bins of variable sizes were used to segment the whole genome. Bin boundaries were decided by the length of reads used in the CNV analysis. For example, simulated reads of length *k* are generated along the whole genome one base pair at a time when the length of reads is *k* bp. Thus, the total number of simulated reads is *L*−*k*+1, where *L* is the length of a chromosome. In our experiments, nearly three billion simulated reads were aligned back to the reference genome and all unique mapping read positions were retained. To divide the whole genome into variable-sized segments, each segment except the last one in each chromosome was possessed to have the same number of uniquely mappable positions. To achieve uniform bins, parameters for bowtie2 in each run were set to be equal. The RD signal was further normalized by locally-weighted polynomial regression (using LOWESS smoother, a function in the R language) and linear interpolation based on the GC content in each bin [3].

**Fig. 2:**
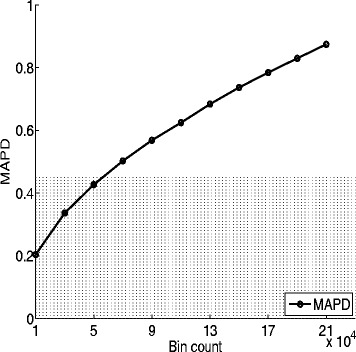
Relationship between bin count (horizontal axis) and MAPD values (vertical axis). With increasing bin count, the quality of the data drops rapidly. A suitable bin count should thus be carefully selected by balancing the high resolution versus good quality as well as large overlap ratio. In our experiments, the maximum tolerable MAPD was set at 0.45 (stippled area) and the bin count was 50000

One of the important issues rising in above procedures is to decide the size of bin count. To investigate its dynamic relationship with the quality of the RC data, a quantitative measurement known as the multiple absolute pairwise difference (MAPD) threshold [7] was used to quantify the data quality. The MAPD is defined as *median*(|log2*x*_*i*+1_− log2*x*_*i*_|), where *x*_*i*_ denotes the RC signal at *i*-th position. A larger MAPD value implies of lower quality of the real RC data and less credibility of the following CNV detection. The relationship between data quality and bin count is shown in Fig. 2. As is evident from the figure, the quality of the RC data drops quickly when the bin count increases. It is due to the RC data tends to be more dispersed (of lower quality) if being preprocessed under a larger bin count.

In analyzing read depth data, a larger bin count is admired to achieve higher resolution. Provided with enough sequencing depth, high resolution analysis makes it accurate in CNV detection. However, if the sequencing depth is low, a larger bin count will deteriorate the data quality and make the data analysis less reliable. In order to find the accurate value in balancing the high resolution versus good quality as well as large overlap ratio *f*(*x*), a simple maximization scheme is defined as following:

$$\max_{x}\left\{(1-{\alpha})g(x)-{\alpha}k(x)\right\} $$

where *g*(·) is a polynomial functions aiming to fitting the *MPAD* function. *k*(·) is the overlap ratio defined in Eq. (1). The parameter of *α* is a trade-off parameter, ranging from 0.20 to 0.30. In our experiments, *α* was set to be 0.208 and it is corresponding to a bin count of 50000. When the bin count is 50000, the ratio between the empirical RC and the one approximated by NB distribution is higher than 90*%* and the MAPD value is closer to the maximum tolerable value (0.45). Therefore, the bin count of 50000 was selected to achieve a nice balance between the data quality and resolution of detection.

### Problem formulation

Mathematically, let ***y***=(*y*_1_,*y*_2_,…,*y*_*n*_) be the observed RD signal in a bin, with ***x***=(*x*_1_,*x*_2_,…,*x*_*n*_) representing the corresponding reconstructed CN. We wish to determine the CN ***x*** that is most likely given by RD ***y***. By Bayes’s Law:

$$P(\boldsymbol{x}|\boldsymbol{y}) =\frac{P(\boldsymbol{y}|\boldsymbol{x})P(\boldsymbol{x})}{P(\boldsymbol{y})}. $$

estimation of the CN ***x*** could be derived by maximizing the posterior probability *P*(***y***|***x***)*P*(***x***). Assuming a negative binomial distribution at each genome position *t* with mean parameter *x* and over-dispersed parameter *α*, we have:

$$P(y_{t}) =\frac{\Gamma(y_{t}+\alpha)}{y_{t}! \Gamma(\alpha)} \left(\frac{ x_{t}}{ x_{t}+\alpha }\right)^{y_{t}} \left(\frac{\alpha}{x_{t}+\alpha }\right)^{\alpha}, $$

where *α* is an over-dispersed parameter that must be estimated empirically. For ease of model derivation, we temporarily assume that its value is known a priori and elaborate its estimation later.

If we assume that the values of *y* at position *t* are independent, then:

$$P(\boldsymbol{y}|\boldsymbol{x}) =\prod_{t} \frac{\Gamma(y_{t}+\alpha)}{y_{t}! \Gamma(\alpha)} \left(\frac{x_{t}}{x_{t}+\alpha}\right)^{y_{t}} \left(\frac{\alpha}{x_{t}+\alpha }\right)^{\alpha}. $$

Considering the characteristics of CNVs, we further require that the prior distribution on CN (after standardization by subtracting its mean value) satisfies assumptions related to two characteristics:

*Smoothness*: CNs at contiguous chromosome positions are similar except for abrupt changes between different segments;

*Sparsity*: CN variants are less common than invariants.

Mathematically, the above two characteristics can be penalized by:

$$P(\boldsymbol{x}) = \exp\left\{-\int(\lambda_{1}|\nabla \boldsymbol{x}| +\lambda_{2}|\boldsymbol{x}|)d\Omega\right\}, $$

where *λ*_1_ and *λ*_2_ are trade-off parameters for respectively controlling the sparsity and smoothness of the CN function. The integration operation takes value along the genome on each bin *Ω*.

Finally, we minimize − log(*P*(***y***|***x***)*P*(***x***)) to seek the maximum posterior probability on ***x***:

2 $$\begin{array}{@{}rcl@{}} \min_{\boldsymbol{x},\alpha}& \left\{-\log \Gamma(\boldsymbol{y}+\alpha)+\log \Gamma(\alpha)-\alpha \log(\alpha)\right.\\  +&\left.(\boldsymbol{y}+\alpha)\log (\boldsymbol{x}+\alpha)^{+}-\boldsymbol{y}\log x^{+}+\lambda_{1}|\nabla \boldsymbol{x}| +\lambda_{2}|\boldsymbol{x}|\vphantom{-\log \Gamma(\boldsymbol{y}+\alpha)+\log \Gamma(\alpha)-\alpha \log(\alpha)}\right\}, \end{array} $$

where *x*^+^= max{0,*x*}. However, minimization of this problem to have optimal *x* and *α* is infeasible because of the presence of the hyperbolic function *Γ*. If one uses the gradient descent method, the computation time needed to approximate the optimal solution will be very large. To alleviate this problem, we use a simple MLE-based method to estimate the value of *α*; thus, Eq. (2) can be simplified as

3 $$ \begin{aligned} &\min_{x} \left\{\sum_{t}\left\{(y_{t}+\alpha)\log (x_{t}+\alpha)^{+}-y_{t}\log x\right\}^{+}\right.\\ &+\left.\int(\lambda_{1}|\nabla x| +\lambda_{2}|x|)d\Omega\right\}, \end{aligned}  $$

where *x*^+^= max{0,*x*}. Once we have obtained the legitimate CN signal, its variants can be easily derived using simple thresholds.

### Numerical solution

The minimization problem (3) is actually a quadratic optimization constrained both by a total variational norm and a *l*_1_ norm. Such minimization problems are widely encountered in various areas, including signal processing and image recovery [23]. Because the optimization problem (3) is convex, multiple standard optimization methods are available for its solution, such as majority minimization [33, 34] and the Lasso approach [11, 35]. Because of the high volume of the sequencing data, however, an efficient numerical solution is desirable for practical usage. This paper proposes to solve Eq. (3) within the framework of the Alternating Direction Method of Multipliers (ADMM) method [23, 35]. The most attractive characteristic of ADMM is its ability to decompose a complex problem into favorably separable subproblems that can then be efficiently solved individually.

Let *g*_1_(***x***)=(***y***+*α*) log(***x***+*α*)−***y*** log***x***, *g*_2_=**1**_+_(·), *g*_3_=*λ*_1_∥·∥_1_, *g*_4_=*λ*_2_∥·−*c*∥_1_, where **1**_+_ is the indicator function for positive real numbers:

$$\mathbf{1}_{+}(x) = \left\{ \begin{array}{ll} 0, & x>0 \\ +\infty, & \text{otherwise}. \end{array} \right. $$

Let ***G***=[***I***;***I***;***∇***;***I***]^*T*^, with ***I*** being the identity matrix and ***∇*** the usual difference matrix. The minimization Eq. (3) can then be accordingly rewritten as:

$$\begin{aligned}[l] \mathbb{L}(\boldsymbol{x})=&\min_{\boldsymbol{x}} {\sum_{t}^{m}}\left\{(y_{t}+\alpha)\log (x_{t}+\alpha)^{+} - y_{t}\log x_{t}^{+} \right.\\ & +\mathbf{1}_{+}(x_{t}))+\lambda_{1}\|\nabla x\|_{1}\\ &+\left.\lambda_{2}\|x_{t}-c\|_{1}\vphantom{(y_{t}+\alpha)\log (x_{t}+\alpha)^{+}}\right\}\\ =&\min_{\boldsymbol{x}} f_{1}(\boldsymbol{x})+f_{2}(\boldsymbol{G}\boldsymbol{x}), \end{aligned} $$

where $f_{1}(\boldsymbol {x})=0,f_{2}(\boldsymbol {x}) = \sum _{j=1}^{4} g_{j}(\boldsymbol {x})$. After introducing a slack variable ***u***=***Gx***, the augmented Lagrange function for $\mathbb {L}(\boldsymbol {x})$ is:

4 $$ \mathbb{L} = \min_{\boldsymbol{x}} f_{1}(\boldsymbol{x})+f_{2}(\boldsymbol{u})+\frac{\boldsymbol{\mu}}{2}\|\boldsymbol{G}\boldsymbol{x}-\boldsymbol{u}\|_{2}^{2},  $$

where ***μ*** is the Lagrange multiplier. The above minimization problem can be now fitted into the ADMM framework and subsequently decoupled into the following two subproblems:

- **Subproblem 1:**$\boldsymbol {x}_{k+1}=\arg \min _{\boldsymbol {x}} f_{1}(\boldsymbol {x})+\frac {\boldsymbol {\mu }}{2}\|\boldsymbol {Gx}-\boldsymbol {u}_{k}-\boldsymbol {d}_{k}\|_{2}^{2}$
- **Subproblem 2:**$\boldsymbol {u}_{k+1}=\arg \min _{\boldsymbol {u}} f_{2}(\boldsymbol {u})+\frac {\boldsymbol {\mu }}{2}\|\boldsymbol {Gx}_{k+1}-\boldsymbol {u}-\boldsymbol {d}_{k}\|_{2}^{2}$
- **Updating:*d***_*k*+1_←***d***_*k*_−(***Gx***_*k*+1_−***u***_*k*+1_).

All that remains are to solving the two subproblems, for which we demonstrate that they can be elegantly solved using standard methods after simple algebraic transformation in Additional file 1: S.2. For the clarity of the numerical scheme, a short introduction of ADMM is also provided in Additional file 1: S.1.

### Parameter pruning

**Fig. 3:**
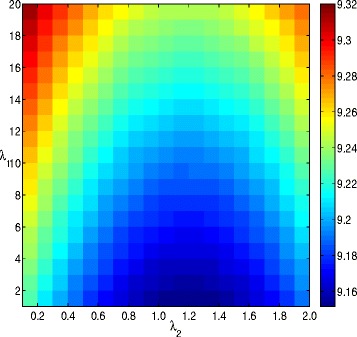
Heatmap of Euclidean distance between the fitter signals and the real copy number signals with respect to the two parameters used by nbCNV. The horizontal axis stands for *λ*_1_ and the vertical axis stands for *λ*_2_. A smaller Euclidean distance implies a better detection performance

The dispersion parameter *α* is associated with the negative binomial distributions of the different CN states. In our experiments, the dispersion parameter was estimated by MLE. In simulation experiments, the RDs from simulated reads of the chromosome-21 sequence without implanted CNVs were used for the MLE estimation of *α*. In empirical experiments, the RD signals from a normal single cell under accession number SRR052047 were employed for estimation of *α*. The parameter *λ*_1_ is used to penalize the total variational term, and *λ*_2_ is used to control the sparsity of the recovered signal. Both of the two parameters were estimated by trials on preliminary experiments. The copy number duplications were implanted artificially in the RD data of SRR052047 by adding one CN to any bins with the duplications. The copy number deletions were generated similarly by subtracting one to any bins overlapped the deletions. It should be noted that SRR052047 was considered as a clean sample (CN=2) and thus its copy number status was known. We run the simluation experiments for different values of *λ*_1_ and *λ*_2_. The Euclidean distance between the fitter signals and the real copy number signals was calculated for evaluating the CNV detection performance. As shown in Fig. 3, one may observe that when the *λ*_1_ was set as 1 and *λ*_2_ was set as 1 the Euclidean distance achieved the minimum. For real data experiments, the two parameters of *λ*_1_ and *λ*_2_ were pruned around 1.

## Results and discussion

### Simulation experiments

**Fig. 4:**
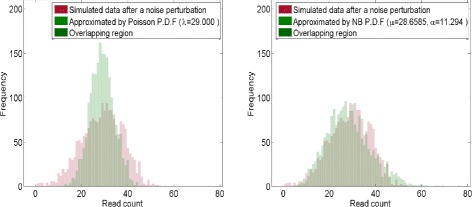
Frequency histograms of contaminated RCs for the SRR052047 in chromosome-21 with implanted CNV sequences. The distribution of contaminated RCs can be better fitted by a negative binomial distribution than a Poisson distribution and is closer to that of empirical data

To evaluate the performance of nbCNV, experiments on a simulation dataset from a chromosome sequence with implanted CNVs were conducted. The chromosome 21 of GRCh37 was used as a template. Variants including duplications and deletions were randomly implanted into it. Our experiments considered only duplications (CN = 3) and deletions (CN = 1) since these two types of CNVs are typically the most challenging problem in distinguishing them from normal CNs. We first doubled the chromosome-21 sequence (CN = 1) to generate the diploid sequence (CN = 2). The length of chromosome 21 without unknown sequences is 35106692 bp and the size of CNVs ranged from 300000 bp to 2000000 bp. For each simulation, 10 CNVs were artificially implanted into the chromosome, from which simulated single-end sequencing reads were created by WgSim [18]. WgSim is a simulation tool to create NGS reads, including single nucleotide polymorphisms, insertion-deletions and sequencing errors from a reference sequence. The simulated reads by WgSim were further contaminated by noises following negative binomial distribution to mimic the technical noises introduced by amplification and sequencing [12]. A total number of 160452 reads with coverage of 0.22 × were generated. Each single-end reads is in 50-bp, similar to the Illumina sequencing platform. As shown in Fig. 4, the frequency histogram of the simulated RCs was approximated by negative binomial and Poisson distribution. One may note that the frequency of the simulated reads was nicely characterized by the negative binomial distribution.

**Fig. 5:**
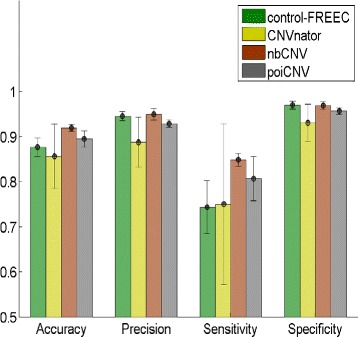
The performance of implanted CNVs detection after four methods of control-FREEC, CNVnator, nbCNV and poiCNV at chromosome-21. Each bar in the plot represents the mean based on 100 simulations of the corresponding measurement as determined by each method. In addition to the error bars, the nbCNV method can clearly be seen to have achieved superior performance in terms of accuracy, precision and sensitivity

To have quantitative comparison on CNV detection, the sampled short reads were aligned back to the reference sequence by bowtie2 [16]. Its output sam files were used as the input for control-FREEC [6], CNVnator [1], nbCNV and our earlier work based on Poisson model named by poiCNV) [32] for performance comparison. As for control-FREEC, the chromosome-21 sequence with implanted CNVs were served as the control sequence. The bin size used in control-FREEC and CNVnator analyses was 50000. The above simulation was run 100 times independently. For quantitative comparison, four measurements including accuracy, precision, sensitivity and specificity were recorded and calculated. Their definitions are as follows:

$$\begin{array}{@{}rcl@{}} Accuracy&=&\frac{TP+TN}{TP+TN+FP+FN} \\ Precision&=&\frac{TP}{TP+FP}\\ Sensitivity&=&\frac{TP}{TP+FN} \\ Specificity&=&\frac{TN}{TN+FP} \end{array} $$

where true positive (TP) is the total number of instances when the CNV regions are correctly identified and true negative (TN) is the number of instances when the normal regions (CN = 2) are detected properly. False positive (FP) and false negative (FN) are defined similarly. The experimental results are summarized in Table 1, in which the best value was highlighted in bold. For visual comparison, a bar graph was also drawn in Fig. 5. Among the 100 simulations, the nbCNV performed superior to poiCNV and its peers by achieving the highest measurements of accuracy, precision and sensitivity. Moreover, nbCNV also resulted in a smaller standard deviations than by control-FREEC and CNVnaotr. Compared with control-FREEC and CNVnaotr, the superior performance of nbCNV is attributed to its effective data preprocessing and robustness in parameter pruning. Compared with poiCNV, the nice performance of nbCNV is attributed to its appropriate noise modelling.

**Table 1:** Quantitative evaluation of the four tested methods in 100 simulation datasets. The best performance was highlighted in bold

Methods	Measurements			
	Accuracy	Precision	Sensitivity	Specificity
CNVnator	85.55±6.56 *%*	88.66±6.69 *%*	74.98±15.96 *%*	92.24±5.39 *%*
nbCNV	**91.83**±**0.98** *%*	**94.85**±**1.05** *%*	**84.78**±**2.17** *%*	96.77±0.69 *%*
control-FREEC	87.59±2.10 *%*	94.43±1.13 *%*	73.32±6.15 *%*	**96.88**±**0.93** *%*
poiCNV	89.41±1.77 *%*	92.76±0.89 *%*	80.61±4.89 *%*	95.58±0.72 *%*

### Application to SCS data from 100 single cells

To further assess the performance of nbCNV in real applications, a SCS dataset from 100 single cells was downloaded from the NCBI SRA under accession number SRP002535 and tested. The original samples were selected from high-grade (III) triple-negative (*ER*^−^, *PR*^−^, *HER*2^−^) ductal carcinomas (T10) [21]. They were preprocessed by flow sorting of single nuclei, whole genome amplification, library construction, and finally sequenced on an Illumina Genome Analyzer [3]. The 100 Illumina runs generated a total of 1.1×10^9^ reads, 5.8×10^10^ base calls (33.3 Gb downloads in sra format) and were thus of low coverage. The data has been used to study the evolutionary dynamics and population structure of tumors in order to have a comprehensive view of the evolutionary process occurring in individual tumor cells [21]. They have been analyzed by fluorescence-activated cell sorting and therefore their ploidy levels were known, including 47 diploids or pseudodiploids (2N), 24 hypodiploids (1.7N) and 29 aneuploids (3N or 3.3N). The diploids or pseudodiploids part consists of cells which have a small number of CNVs as a whole, while the hypodiploids part shows narrow deletions and the aneuploids part shows numerous copy number duplications [21]. The ploidy information could serve as benchmark information for evaluating the clustering performance in these 100 cells.

**Fig. 6:**
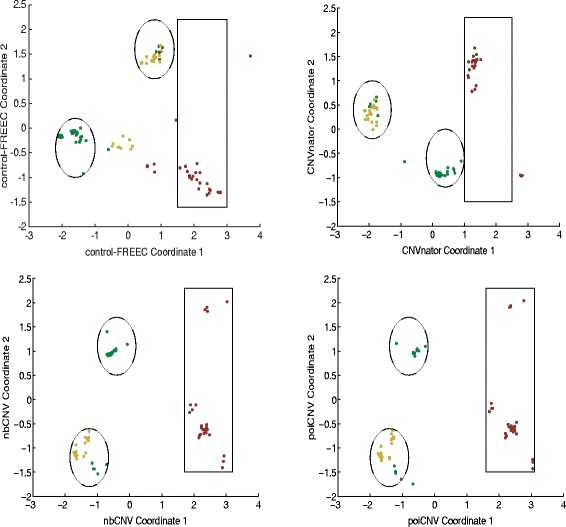
Multidimensional scaling of 100 single cells. Diploid (2N), hypodiploid (1.7N) and aneuploid (3N or 3.3N) fractions are shown in green, yellow and red, respectively. Clustering results after nbCNV were better than those after CNVnator, when comparing the number of covered dots. Compared with the other three methods, control-FREEC resulted in a smaller inter-cluster distance between diploid and hypodiploid fractions, and thus was less satisfactory

**Fig. 7:**
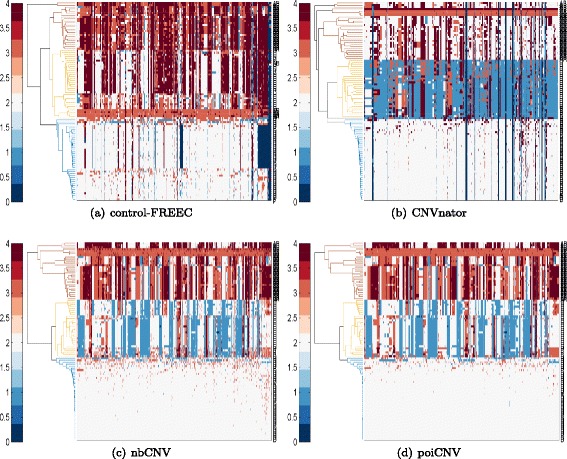
Results of hierarchical clustering and heat map of copy number profiles of 100 single cells after **a** control-FREEC, **b** CNVnator, **c** nbCNV and **d** poiCNV analysis. Different colors are used to distinguish the generated clusters. A comparative evaluation of the performance of the different detection methods based on the clustering results is given in Table 2

**Fig. 8:**
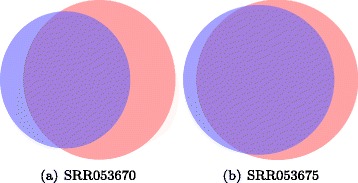
Wayne comparison on detected CNVs by nbCNV and poiCNV on two samples. The pink part measures the number of CNVs detected by nbCNV while the purple represents poiCNV and the overlay region represents both. It implies of a possibly better coverage yet higher false positive by nbCNV

The proposed nbCNV as well as the other three methods of poiCNV, CNVnator and control-FREEC were applied on the sequence data to have their CN profiles. For visual comparison, multidimensional scaling (MDS) was performed by mapping each sample from the high-dimensional space to a visually acceptable one (i.e., two dimensions). The diploid (2N), hypodiploid (1.7N) and aneuploid (3N or 3.3N) fractions are highlighted in Fig. 6. Five diploid cells were mistakenly classified into the hypodiploid fraction by nbCNV and poiCNV. However, when we retrospectively examined the five diploid cells, we found that they possess abundant CNVs compared with other diploid cells and thus making it difficult to be merged with others. The clustering result on the CN profiles after nbCNV was better than the one by CNVnator by covering most of the sample dots. The one after control-FREEC method resulted in a smaller inter-cluster distance between diploid and hypodiploid fractions. To further visualize the evolutionary history of the 100 single cells, hierarchical clustering was computed and shown in Fig. 7. The misclassified cell number after each method was summarized in Table 2. The proposed nbCNV and poiCNV achieved superior performance by only misclassifying five cells. In comparison, CNVnator misclassified 6 cells and control-FREEC misclassified 10 cells. It should be noted that although ploidy information could serve as benchmark information for evaluating the clustering results. However, using clustering accuracy to evaluate the performance of methods was coarse-grained. For example, nbCNV and poiCNV were shown to perform equally well in term of MDS and clustering accuracy. Since the real copy number profiles of these sequence data were unknown, we pictured the number of detected CNVs after nbCNV and poiCNV by Wayne chart at two typical cells (SRR053670, SRR053675). As shown in Fig. 8, nbCNV can detect more CNVs than by poiCNV. It implies a possible good coverage yet higher false positive. We also reported the detection results on other cells by nbCNV and poiCNV in Additional file 1: S.3.

**Table 2:** Quantitative evaluation of the four copy number variant detection methods based on clustering of single-cell sequencing data from 100 cells

Methods	Classification error count		
	cluster 1	cluster 2	cluster 3
CNVnator	0	6	0
nbCNV	0	5	0
control-FREEC	0	10	0
poiCNV	0	5	0

## Conclusions

We have presented a RD-based method to detect CNVs from over-dispersed sequencing data such as SCS data. Taking into account the over-dispersed noise in the SCS data and the characteristics of CNV patterns, the method uses negative binomial distributions to model the RD signal and imposes sparsity and smoothness constraints to transform CNV detection into a quadratic optimization problem. Comparative experiments with other CNV detection methods on simulated data and an empirical SCS dataset demonstrated that our method is superior in terms of accuracy, precision and sensitivity for CNV detection. Compared with other methods, the robustness in parameter pruning in our CNV detection method contributes to a more steady performance.

## Additional file

Additional file 1 Contains a formal description of the ADMM algorithm and the CNV detection results on SCS ductal carcinomas (T10) data by nbCNV and poiCNV. (PDF 166 kb)

## Abbreviations

CNVs: Copy number variants
CN: Copy number
SCS: Single-cell sequencing
WGA: Whole-genome amplification
RD: Read depth
GC: Guanine-cytosine
CBS: Circular binary segmentation
ADMM: Alternating direction minimization method
MAPD: Multiple absolute pairwise difference
