# Supplementary material for: nbCNV: a multi-constrained optimization model for discovering copy number variants in single-cell sequencing data
Source: BMC Bioinformatics. 2016 Sep 17;17:384. doi: 10.1186/s12859-016-1239-7 (PMC5027123; doi:10.1186/s12859-016-1239-7)
Supplement: Additional file 1 — Contains a formal description of the ADMM algorithm and the CNV detection results on SCS ductal carcinomas (T10) data by nbCNV and poiCNV. (PDF 166 kb) [file 12859_2016_1239_MOESM1_ESM.pdf]

## S1 Alternating Direction Method of Multipliers and Its Convergence

The Alternating Direction Method of Multipliers (ADMM) is designed to solve a generally well-structured optimization problem,

$$\min f_1(\boldsymbol{x}) + f_2(\boldsymbol{G}\boldsymbol{x}) \tag{1}$$

where $f_1 : \mathbb{R}^d \to \mathbb{R}$ and $f_2 : \mathbb{R}^p \to \mathbb{R}$ are convex functions. $\boldsymbol{G} \in \mathbb{R}^{p\times d}$ is a transformation matrix with full column rank.

Given a Lagrangian multiplier $\boldsymbol{d}$ and a slack variable $\boldsymbol{u} = \boldsymbol{G}\boldsymbol{x}$, the augmented Lagrangian function for the above function is,

$$\mathrm{L}(\boldsymbol{x}, \boldsymbol{u}, \boldsymbol{d}) = f_1(\boldsymbol{x}) + f_2(\boldsymbol{u}) + < \boldsymbol{d}, \boldsymbol{G}\boldsymbol{x} - \boldsymbol{u} > + \frac{\boldsymbol{\mu}}{2}\|\boldsymbol{G}\boldsymbol{x} - \boldsymbol{u}\|_2^2 \tag{2}$$

The above minimization problem could be addressed solved by alternating solving the following subproblems sequentially:,

$$\begin{cases} \boldsymbol{x}^{k+1} & \in \arg\min \mathrm{L}(\boldsymbol{x}, \boldsymbol{u}^k, \boldsymbol{d}^k) \\ \boldsymbol{u}^{k+1} & \in \arg\min \mathrm{L}(\boldsymbol{x}^{k+1}, \boldsymbol{u}, \boldsymbol{d}^k) \\ \boldsymbol{d}^{k+1} & = \boldsymbol{d}^k - \beta(\boldsymbol{G}\boldsymbol{x}^{k+1} - \boldsymbol{u}^{k+1}). \end{cases} \tag{3}$$

**Theorem 1 (Eckstein-Bertsekas** [1] Consider problem (1), $f_1 : \mathbb{R}^d \to \mathbb{R}$ and $f_2 : \mathbb{R}^p \to \mathbb{R}$ are closed, proper, convex functions. For arbitrary $\beta > 0$ and $\boldsymbol{u}_0, \lambda_0 \in \mathbb{R}^p$, if there exist two convergent sequences of $\{\eta^k \geq 0, k = 0, 1, \cdots\}$ and $\{\rho^k \geq 0, k = 0, 1, \cdots\}$, such that there are three sequences $\{\boldsymbol{x}^k \in \mathbb{R}^d, k = 0, 1, \cdots\}$,$\{\boldsymbol{u}^k \in \mathbb{R}^p, k = 0, 1, \cdots\}$,$\{\boldsymbol{d}^k \in \mathbb{R}^p, k = 0, 1, \cdots\}$ that satisfy

$$\|\boldsymbol{x}^{k+1} - \arg\min_{\boldsymbol{x}} f_1(\boldsymbol{x}) + \frac{\mu}{2}\|\boldsymbol{G}\boldsymbol{x} - \boldsymbol{u}^{k+1} - \boldsymbol{d}^k\|_2^2\| \leq \eta^k$$
$$\|\boldsymbol{u}^{k+1} - \arg\min_{\boldsymbol{u}} f_2(\boldsymbol{u}) + \frac{\mu}{2}\|\boldsymbol{G}\boldsymbol{x}^{k+1} + \boldsymbol{u} - \boldsymbol{d}^k\|_2^2\| \leq \rho^k$$
$$\boldsymbol{d}^{k+1} = \boldsymbol{d}^k - \beta(\boldsymbol{G}\boldsymbol{x}^{k+1} - \boldsymbol{u}^{k+1})$$

Then, if Eq. (1) has a solution $\boldsymbol{x}^*$, it follows that $\boldsymbol{x}^k \to \boldsymbol{x}^*$.

## S2 Numerical Solution

The minimization problem (4) in the manuscript can be fitted into ADMM framework and subsequently decoupled into the following two subproblems:

**Subproblem 1:** $\boldsymbol{x}_{k+1} = \arg\min_{\boldsymbol{x}} f_1(\boldsymbol{x}) + \frac{\boldsymbol{\mu}}{2}\|\boldsymbol{G}\boldsymbol{x} - \boldsymbol{u}_k - \boldsymbol{d}_k\|_2^2$

**Subproblem 2:** $\boldsymbol{u}_{k+1} = \arg\min_{\boldsymbol{u}} f_2(\boldsymbol{u}) + \frac{\boldsymbol{\mu}}{2}\|\boldsymbol{G}\boldsymbol{x}_{k+1} - \boldsymbol{u} - \boldsymbol{d}_k\|_2^2$

**Updating:** $\boldsymbol{d}_{k+1} \leftarrow \boldsymbol{d}_k - \beta(\boldsymbol{G}\boldsymbol{x}_{k+1} - \boldsymbol{u}_{k+1})$.

All that remains is the solution of the two subproblems, which we now demonstrate can be elegantly solved using standard methods after simple algebraic transformation.

Regarding **subproblem 1**, one can verify that:

$$\begin{aligned} \boldsymbol{x}_{k+1} & \in \arg\min_{\boldsymbol{x}} \frac{\boldsymbol{\mu}}{2}\|\boldsymbol{G}\boldsymbol{x} - \boldsymbol{u}_k - \boldsymbol{d}_k\|_2^2 \\ & = (\boldsymbol{G}^T\boldsymbol{G})^{-1}\boldsymbol{G}^T\boldsymbol{\xi}_k \\ & = (3\boldsymbol{I} + \boldsymbol{D}^T\boldsymbol{D})^{-1}(\boldsymbol{\xi}_1 + \boldsymbol{D}^T\boldsymbol{\xi}_2 + \boldsymbol{\xi}_3 + \boldsymbol{\xi}_4), \end{aligned}$$

where $\boldsymbol{\xi}_k = \boldsymbol{u}_k + \boldsymbol{d}_k$, while $\boldsymbol{D}$ is the total variational difference matrix. Thus, $\boldsymbol{D}^T\boldsymbol{D}$ is a circular matrix and can thus be diagonalized by the discrete Fourier transform. By a series of simple calculations, the analytical solution for subproblem 1 is given by:

$$\boldsymbol{x}_{k+1}^* = \boldsymbol{F}^T(3\boldsymbol{I} + \boldsymbol{K}^2)^{-1}(3\boldsymbol{I} + \boldsymbol{K})\boldsymbol{F}(\boldsymbol{\xi}_1 + \boldsymbol{D}^T\boldsymbol{\xi}_2 + \boldsymbol{\xi}_3 + \boldsymbol{\xi}_4), \tag{4}$$

where $\boldsymbol{F}$ is the 2-D discrete Fourier transformation matrix and $\boldsymbol{K} = \frac{diag(FD)}{n}$.

The solution to **subproblem 2** amounts to solving:

$$\boldsymbol{u}_{k+1} \in \arg\min_{\boldsymbol{u}} f_2(\boldsymbol{u}) + \frac{\boldsymbol{\mu}}{2}\|\boldsymbol{G}\boldsymbol{x}_{k+1} - \boldsymbol{u} - \boldsymbol{d}_k\|_2^2.$$

This equation can be further decoupled with respect to each $\boldsymbol{u}^{(i)}$ in $\boldsymbol{u} = [\boldsymbol{u}^{(1)}, \boldsymbol{u}^{(2)}, \boldsymbol{u}^{(3)}, \boldsymbol{u}^{(4)}]^T$ for $i = 1, 2, 3, 4$, with each subpart then solved separately. The decomposed problems are of the form:

$$\boldsymbol{u}_{k+1}^{(j)} \leftarrow \arg\min_{\boldsymbol{v}} g_j(\boldsymbol{v}) + \frac{\boldsymbol{\mu}}{2}\|\boldsymbol{v} - \boldsymbol{s}_k^{(j)}\|_2^2, \tag{5}$$

for $j = 1, \ldots, 4$, with $\boldsymbol{s}_k^{(j)} = (\boldsymbol{G}\boldsymbol{x}_{k+1})^{(j)} - \boldsymbol{d}_k^{(j)}$.

For $i = 1$, the minimization problem (5) has the form:

$$\arg\min_{\boldsymbol{v}}\{\boldsymbol{y}\log\boldsymbol{v} - (\boldsymbol{y} + \boldsymbol{\alpha})\log(\boldsymbol{v} + \boldsymbol{\alpha}) + \frac{\boldsymbol{\mu}}{2}(\boldsymbol{v} - \boldsymbol{\eta})^2\}. \tag{6}$$

This turns out to be a cubic algebraic equation and its solution is elegantly given by the Shengjing Formula. For $i = 2$, the minimization problem (5) has the form:

$$\arg\min_{\boldsymbol{v}}\{\mathbf{1}_+(\boldsymbol{v}) + \frac{\boldsymbol{\mu}}{2}(\boldsymbol{v} - \boldsymbol{\eta})^2\}. \tag{7}$$

Its solution is given by a hard threshold on $\eta$:

$$\boldsymbol{v}^* = \max\{\boldsymbol{\eta}, 0\}. \tag{8}$$

For $i = 3$, the minimization problem (5) has the form:

$$\arg\min_{\boldsymbol{v}}\{\lambda_1\|\boldsymbol{v}\|_1 + \frac{\boldsymbol{\mu}}{2}(\boldsymbol{v} - \boldsymbol{\eta})^2\}.$$

Its solution is given by the Moreau proximity operator, which is a simple soft threshold:

$$\begin{aligned}\boldsymbol{v}^* &= soft(\boldsymbol{\eta}, \frac{\lambda_1}{\boldsymbol{\mu}}) \\ &= sign(\boldsymbol{v})\max\{|\boldsymbol{\eta}| - \frac{\lambda_1}{\boldsymbol{\mu}}, 0\}.\end{aligned}$$

For $i = 4$, the minimization problem (5) has the form:

$$\arg\min_{\boldsymbol{v}}\{\lambda_2\|\boldsymbol{v} - c\|_1 + \frac{\boldsymbol{\mu}}{2}(\boldsymbol{v} - \boldsymbol{\eta})^2\}.$$

Its solution is also given by the Moreau proximity operator:

$$\boldsymbol{v}^* = soft(\boldsymbol{\eta} - c, \frac{\lambda_2}{\boldsymbol{\mu}}). \quad (9)$$

Finally, the auxiliary sequences of $\{\boldsymbol{d}_k\}$ are updated by:

$$\boldsymbol{d}_{k+1} \leftarrow \boldsymbol{d}_k - \beta(\boldsymbol{G}\boldsymbol{x}_{k+1} - \boldsymbol{u}_k). \quad (10)$$

Once the CN from the raw RD signals has been successfully reconstructed, application of a simple threshold can be used to find the variants.

## S3 list of the CNV detection results on SCS data [2] by nbCNV and poiCNV

In the experiment on 100 SCS dataset [2], we have noted that using cluster accuracy to evaluate the detection results was coarse-grained. The model of nbCNV and poiCNV were shown to perform equally well in term of MDS and clustering accuracy. For though analysis, we summarized the CNV detection results on the 100 cell in Table 1 for 24 hypodiploids (1.7N) cells, Table. 2 for 47 diploids or pseudodiploids (2N) cells and Table 3 for 29 aneuploids (3N or 3.3N) cells. The first column is the cell id. The second and third column represent the number of CNVs detected by poiCNV and by nbCNV, respectively. The third column counts the number of common CNVs detected by the twos simultaneously. The value within round brackets measures the ratio between the number of CNVs and the total number of CNVs for each cells. The number of CNVs is defined as the number of bins which were detected as variations.

**Table 1 CNV detection results on 24 hypodiploids (1.7N) cells. The value within round brackets measures the ratio between the number of CNVs and the total number of CNVs for each cells.**

| id | poi | common | nb |
|---|---|---|---|
| SRR053681 | 193(0.73%) | 25857(97.23%) | 544(2.05%) |
| SRR054213 | 84(0.31%) | 26523(98.81%) | 236(0.88%) |
| SRR054565 | 1103(3.74%) | 27229(92.30%) | 1169(3.96%) |
| SRR054566 | 99(0.37%) | 26155(97.11%) | 678(2.52%) |
| SRR054567 | 92(0.38%) | 23917(97.72%) | 466(1.90%) |
| SRR054568 | 96(0.36%) | 26195(98.40%) | 330(1.24%) |
| SRR054569 | 28(0.11%) | 24691(99.26%) | 156(0.63%) |
| SRR054570 | 94(0.36%) | 26006(98.28%) | 362(1.37%) |
| SRR054571 | 151(0.56%) | 25870(96.32%) | 837(3.12%) |
| SRR054572 | 1108(3.81%) | 26936(92.51%) | 1074(3.69%) |
| SRR054573 | 189(0.71%) | 25941(96.98%) | 618(2.31%) |
| SRR054574 | 136(0.49%) | 27016(98.00%) | 416(1.51%) |
| SRR054575 | 100(0.36%) | 27460(98.48%) | 325(1.17%) |
| SRR054576 | 81(0.33%) | 24677(99.24%) | 108(0.43%) |
| SRR054577 | 130(0.46%) | 27735(98.90%) | 179(0.64%) |
| SRR054578 | 149(0.52%) | 28098(98.35%) | 322(1.13%) |
| SRR054592 | 1257(4.99%) | 22109(87.81%) | 1812(7.20%) |
| SRR054594 | 807(3.16%) | 23236(90.94%) | 1508(5.90%) |
| SRR054596 | 883(3.29%) | 24035(89.56%) | 1919(7.15%) |
| SRR054597 | 563(2.26%) | 21977(88.26%) | 2360(9.48%) |
| SRR054598 | 1377(5.50%) | 21604(86.23%) | 2072(8.27%) |
| SRR054599 | 1374(5.60%) | 21116(86.02%) | 2059(8.39%) |
| SRR054600 | 829(3.34%) | 22371(90.24%) | 1590(6.41%) |
| SRR054601 | 667(2.53%) | 24336(92.45%) | 1321(5.02%) |
| SRR054602 | 205(0.57%) | 35858(99.22%) | 77(0.21%) |

**Table 2 CNV detection results on 47 diploids or pseudodiploids (2N) cells. The value within round brackets measures the ratio between the number of CNVs and the total number of CNVs for each cells.**

| id | poi | common | nb |
|---|---|---|---|
| SRR052047 | 3(0.27%) | 86(7.75%) | 1021(91.98%) |
| SRR052148 | 0(0.00%) | 0(0.00%) | 90(100.00%) |
| SRR053437 | 0(0.00%) | 36(10.84%) | 296(89.16%) |
| SRR053600 | 543(10.80%) | 1694(33.68%) | 2792(55.52%) |
| SRR053602 | 0(0.00%) | 62(31.31%) | 136(68.69%) |
| SRR053604 | 237(8.38%) | 428(15.13%) | 2163(76.49%) |
| SRR053605 | 0(0.00%) | 31(16.40%) | 158(83.60%) |
| SRR053606 | 1(0.09%) | 173(14.76%) | 998(85.15%) |
| SRR053607 | 7(1.63%) | 33(7.67%) | 390(90.70%) |
| SRR053608 | 4(1.57%) | 33(12.99%) | 217(85.43%) |
| SRR053609 | 0(0.00%) | 121(20.58%) | 467(79.42%) |
| SRR053610 | 0(0.00%) | 0(0.00%) | 139(100.00%) |
| SRR053611 | 0(0.00%) | 0(0.00%) | 26(100.00%) |
| SRR053615 | 0(0.00%) | 0(0.00%) | 24(100.00%) |
| SRR053616 | 0(0.00%) | 0(0.00%) | 5(100.00%) |
| SRR053617 | 31(25.83%) | 0(0.00%) | 89(74.17%) |
| SRR053618 | 36(24.66%) | 0(0.00%) | 110(75.34%) |
| SRR053619 | 0(0.00%) | 0(0.00%) | 103(100.00%) |
| SRR053620 | 0(0.00%) | 0(0.00%) | 398(100.00%) |
| SRR053623 | 0(0.00%) | 35(26.52%) | 97(73.48%) |
| SRR053624 | 0(0.00%) | 0(0.00%) | 222(100.00%) |
| SRR053629 | 65(5.58%) | 104(8.93%) | 995(85.48%) |
| SRR053630 | 0(0.00%) | 0(0.00%) | 313(100.00%) |
| SRR053631 | 0(0.00%) | 64(9.58%) | 604(90.42%) |
| SRR053632 | 81(5.63%) | 207(14.39%) | 1150(79.97%) |
| SRR053633 | 1(0.41%) | 53(21.72%) | 190(77.87%) |
| SRR053634 | 0(0.00%) | 0(0.00%) | 72(100.00%) |
| SRR053635 | 47(12.34%) | 77(20.21%) | 257(67.45%) |
| SRR053636 | 44(6.17%) | 236(33.10%) | 433(60.73%) |
| SRR053637 | 0(0.00%) | 0(0.00%) | 112(100.00%) |
| SRR053638 | 1(0.13%) | 39(5.01%) | 738(94.86%) |
| SRR053639 | 35(1.27%) | 672(24.30%) | 2059(74.44%) |
| SRR053666 | 10(0.55%) | 605(33.43%) | 1195(66.02%) |
| SRR053667 | 167(5.49%) | 504(16.56%) | 2372(77.95%) |
| SRR053668 | 2211(8.59%) | 13114(50.96%) | 10410(40.45%) |
| SRR053669 | 0(0.00%) | 0(0.00%) | 228(100.00%) |
| SRR053670 | 2188(12.55%) | 9041(51.85%) | 6208(35.60%) |
| SRR053671 | 1785(8.19%) | 17421(79.89%) | 2601(11.93%) |
| SRR053672 | 1463(11.77%) | 5346(42.99%) | 5626(45.24%) |
| SRR053673 | 735(9.22%) | 3619(45.41%) | 3615(45.36%) |
| SRR053674 | 31(1.40%) | 429(19.35%) | 1757(79.25%) |
| SRR053675 | 317(7.66%) | 3011(72.80%) | 808(19.54%) |
| SRR053676 | 6829(15.45%) | 35213(79.65%) | 2166(4.90%) |
| SRR053677 | 2425(6.39%) | 30835(81.23%) | 4699(12.38%) |
| SRR053678 | 1596(4.86%) | 27758(84.47%) | 3506(10.67%) |
| SRR053679 | 2606(7.12%) | 30958(84.61%) | 3026(8.27%) |
| SRR053680 | 3562(8.94%) | 31934(80.15%) | 4345(10.91%) |

**Table 3 CNV detection results on 29 aneuploids (3N or 3.3N) cells. The value within round brackets measures the ratio between the number of CNVs and the total number of CNVs for each cells.**

| id | poi | common | nb |
|---|---|---|---|
| SRR054602 | 205(0.57%) | 35858(99.22%) | 77(0.21%) |
| SRR054603 | 100(0.28%) | 34838(99.28%) | 151(0.43%) |
| SRR054604 | 1602(4.16%) | 36507(94.84%) | 383(1.00%) |
| SRR054605 | 175(0.48%) | 35672(98.38%) | 413(1.14%) |
| SRR054606 | 499(1.42%) | 34384(97.85%) | 258(0.73%) |
| SRR054607 | 633(1.77%) | 34671(97.10%) | 401(1.12%) |
| SRR054608 | 186(0.52%) | 35415(99.12%) | 128(0.36%) |
| SRR054609 | 101(0.29%) | 33988(99.11%) | 204(0.59%) |
| SRR054610 | 59(0.16%) | 35640(99.17%) | 241(0.67%) |
| SRR054611 | 43(0.12%) | 34372(98.99%) | 308(0.89%) |
| SRR054612 | 189(0.55%) | 34378(99.34%) | 41(0.12%) |
| SRR054613 | 659(1.78%) | 36192(97.95%) | 100(0.27%) |
| SRR054614 | 55(0.15%) | 35329(99.16%) | 244(0.68%) |
| SRR054615 | 73(0.21%) | 35417(99.64%) | 55(0.15%) |
| SRR054616 | 42(0.12%) | 36004(99.66%) | 80(0.22%) |
| SRR054618 | 192(0.54%) | 35274(98.93%) | 190(0.53%) |
| SRR054620 | 248(0.69%) | 35770(99.24%) | 26(0.07%) |
| SRR054622 | 120(0.33%) | 36097(99.01%) | 242(0.66%) |
| SRR054626 | 191(0.57%) | 30850(91.86%) | 2543(7.57%) |
| SRR054632 | 261(0.80%) | 31442(96.19%) | 986(3.02%) |
| SRR054633 | 180(0.56%) | 31325(96.84%) | 841(2.60%) |
| SRR054634 | 131(0.39%) | 32892(97.08%) | 859(2.54%) |
| SRR089377 | 141(0.28%) | 49311(99.64%) | 39(0.08%) |
| SRR089378 | 221(0.45%) | 49298(99.55%) | 0(0.00%) |
| SRR089379 | 458(0.93%) | 48994(98.98%) | 45(0.09%) |
| SRR089397 | 4(0.01%) | 49663(99.99%) | 0(0.00%) |
| SRR089401 | 64(0.21%) | 29438(97.04%) | 834(2.75%) |
| SRR089402 | 29(0.10%) | 29505(99.47%) | 127(0.43%) |
| SRR089403 | 36(0.12%) | 28951(98.99%) | 260(0.89%) |

**Author details**

**References**

1. J. Eckstein and D. P. Bertsekas. On the douglasrachford splitting method and the proximal point algorithm for maximal monotone operators. *Mathematical Programming*, 55(1-3):293–318, 1992.
2. N. Navin, J. Kendall, J. Troge, P. Andrews, L. Rodgers, J. McIndoo, K. Cook, A. Stepansky, D. Levy, D. Esposito, L. Muthuswanmy, A. Kransnitz, W. McCombie, J. Hicks, and M. Wigler. Tumour evolution inferred by single-cell sequencing. *Nature*, 472(7341):90–94, 2011.
